# Anxiolytic Activity and Brain Modulation Pattern of the α-Casozepine-Derived Pentapeptide YLGYL in Mice

**DOI:** 10.3390/nu12051497

**Published:** 2020-05-21

**Authors:** Simon Benoit, Catherine Chaumontet, Jessica Schwarz, Céline Cakir-Kiefer, Audrey Boulier, Daniel Tomé, Laurent Miclo

**Affiliations:** 1 INRAE, URAFPA, Université de Lorraine, F-54000 Nancy, France; celine.cakir-kiefer@univ-lorraine.fr (C.C.-K.); Laurent.Miclo@univ-lorraine.fr (L.M.); 2 UMR PNCA, AgroParisTech, Inrae, Université Paris-Saclay, F-75231 Paris, France; catherine.chaumontet@agroparistech.fr (C.C.); daniel.tome@agroparistech.fr (D.T.); 3 Ingredia, F-62000 Arras, France; jessica.schwarz@outlook.com (J.S.); a.boulier@ingredia.com (A.B.); 4 CALBINOTOX, Université de Lorraine, F-54000 Nancy, France

**Keywords:** milk bioactive peptide, α-Casozepine, proteolytic fragments, anxiety, neuronal activity modulation, c-Fos

## Abstract

α-Casozepine (α-CZP) is an anxiolytic-like bioactive decapeptide derived from bovine α_s1_-casein. The N-terminal peptide YLGYL was previously identified after proteolysis of the original peptide in an in vitro digestion model. Its putative anxiolytic-like properties were evaluated in a Swiss mice model using a light/dark box (LDB) after an intraperitoneal injection (0.5 mg/kg). The effect of YLGYL on c-Fos expression in brain regions linked to anxiety regulation was afterwards evaluated via immunofluorescence and compared to those of α-CZP and diazepam, a reference anxiolytic benzodiazepine. YLGYL elicited some anxiolytic-like properties in the LDB, similar to α-CZP and diazepam. The two peptides displayed some strong differences compared with diazepam in terms of c-Fos expression modulation in the prefontal cortex, the amygdala, the nucleus of the tractus solitarius, the periaqueductal grey, and the raphe magnus nucleus, implying a potentially different mode of action. Additionally, YLGYL modulated c-Fos expression in the amygdala and in one of the raphe nuclei, displaying a somewhat similar pattern of activation as α-CZP. Nevertheless, some differences were also spotted between the two peptides, making it possible to formulate the hypothesis that these peptides could act differently on anxiety regulation. Taken together, these results showed that YLGYL could contribute to the in vivo overall action of α-CZP.

## 1. Introduction

α-Casozepine (α-CZP), a decapeptide corresponding to the fragment 91–100 (YLGYLEQLLR) of bovine milk α_s1_-casein, appears as the component carrying the anxiolytic-like activity of a tryptic hydrolysate of bovine α_s1_-casein [1]. The properties of this hydrolysate are close to those of the benzodiazepine (BZDs) family despite not showing the associated side effects of these drugs, such as habituation or sedation [2].

The anxiolytic-like effects of the tryptic hydrolysate of bovine α_s1_-casein were highlighted after intraperitoneal (IP) and *per os* administration in rodents, cats, dogs, horses, and ponies [1,3,4,5,6,7,8]. The hydrolysate also demonstrated anticonvulsant [1] and sleep-protecting effects in rodents [9,10]. Clinical trials revealed that it had a positive influence on both physical and psychological anxiety symptoms in healthy and stressed human subjects [11,12,13]. α-CZP also displayed anxiolytic-like activities in rats [1,14] and mice [15,16] and modulated neuronal activity in several brain regions linked to anxiety regulation in mice [16]. Studies of α-CZP digestion in in vitro models revealed that the four peptide bonds of the N-terminal part of the peptide showed some resistance toward hydrolysis by the gastric and pancreatic proteases tested (pepsin, chymotrypsin, Corolase PP^®^). The main peptide recovered in the medium corresponded to the fragment 91–97 of bovine α_s1_-casein (YLGYLEQ), and the fragment 91–95 (YLGYL) was recovered to a lesser extent [14]. The peptide α_s1_-CN-(f91–97) displayed anxiolytic-like effects in rats after IP administration in three behavioural tests [14] and its absorption through a Caco2 monolayer was facilitated by the presence of bile salts [17]. The search for the actual peptide, or actual peptides, carrying the anxiolytic-like properties of the tryptic hydrolysate of bovine milk α_s1_-casein remains.

In this context, we proposed to evaluate the anxiolytic-like properties of the other fragment obtained after the in vitro digestion of α-CZP, YLGYL. Furthermore, as α-CZP was shown to modify neuronal activation in the brain regions related to anxiety regulation [16], we also wanted to evaluate the ability of this shorter peptide to modulate brain activity in these same regions. The present study was then designed to evaluate YLGYL in vivo anxiolytic-like properties and its action on brain activity by evaluating the expression of c-Fos, a neuronal activation marker [18,19]. Focus was set on brain areas involved in anxiety regulation, and more specifically on brain regions where differences had already been spotted between α-CZP and diazepam, i.e., the prefrontal cortex and amygdala [16], as both of these regions have been well described regarding their role in anxiety regulation [20,21], and both are relevant when studying potential anxiolytic molecules [22].

## 2. Materials and Methods

### 2.1. Studied Compounds and Peptides

α-CZP (1 mg/kg, i.e., 0.8 µmol/kg) and YLGYL (0.5 mg/kg, i.e., 0.8 µmol/kg) both from Genosphere Biotechnologies, France, and diazepam (1 mg/kg, i.e., 3.5 µmol/kg, Valium, Roche, Switzerland) were diluted in a vehicle aqueous solution of 0.2% methylcellulose and 1% glycerol. The dose of intraperitoneally administered α-CZP that displays effects comparable to those of diazepam is approximately 2.5 times higher in mice than in rats (0.8 μmol/kg [16] compared to 0.32 μmol/kg [1]). Studies with the oral administration of α_s1_-casein tryptic hydrolysate in various species used an α-CZP dose ranging from about 0.03 μmol/kg (horse [5]) to about 0.22 μmol/kg (rat [8,9], cat [3]). A dose of pure synthetic α-CZP displaying an anxiolytic activity in mice after oral administration was higher, i.e., 3.5 μmol/kg [15]. The effective dose of α-CZP seems lower when the latter is within the hydrolysate. The dose of YLGYL was therefore chosen according to the animal model and the route of administration and was fixed at 0.8 μmol/kg. Each compound was injected intraperitoneally, in a volume of 5 mL/kg body weight. The control group received the same volume of the vehicle.

### 2.2. Animals

Forty-eight, 9-week-old, male Swiss mice (Janvier Labs, Le Genest-Saint-Isle, France), split into two distinct batches of 32 and 16 animals for the two experiments detailed below, were single-housed and maintained in a controlled environment (temperature 22 ± 1 °C, humidity 60%) with a 12 h reversed light cycle (lights off from 08:00 to 20:00). After a week of acclimation, all animals received a soy protein-based diet [16] to exclude a potential in vivo formation of casein-derived bioactive peptides. The animals had continuous access to water and food during all the experiments and their active period was chosen to conduct the behavioural tests. The French Ministère de l’Agriculture, de l’Agroalimentaire et de la Forêt approved all protocols following the Comité d′Éthique en Expérimentation Animale of Jouy-en-Josas recommendation (N°02237.01).

### 2.3. Experiment 1: Evaluation of Anxiolytic-Like Properties of YLGYL

To assess the anxiolytic-like properties of YLGYL before evaluating its potential neuronal activation with immunofluorescence, an initial batch of 32 animals was used in a light/dark box (LDB) device. The LDB apparatus, experimental conditions and procedure, and collected data were defined according to a previous experiment [16]. Mice were placed in the LDB, 30 min after an IP injection (5 mL/kg body weight) of either the vehicle, α-CZP (1 mg/kg), YLGYL (0.5 mg/kg), or diazepam (1 mg/kg) (*n* = 8/group) and their behaviours were recorded for 5 min. All data were analysed before the second experiment described below was started.

### 2.4. Experiment 2: Evaluation of YLGYL Effects on Neuronal Activation

A second batch of 16 naive mice was used to evaluate the effects of YLGYL on neuronal activation using c-Fos. The methods for neuronal activation evaluation using c-Fos immunofluorescence followed our previously described procedure [16], but this time, only the anxiety-inducing situation was used as it is mandatory to induce the modulation of c-Fos immunostaining by α-CZP. Briefly, two weeks after acclimation to the lab conditions, this batch of animals received an IP injection (5 mL/kg body weight) of either the vehicle, α-CZP (1 mg/kg), YLGYL (0.5 mg/kg), or diazepam (1 mg/kg) (*n* = 4/group). Thirty minutes later, the animals were placed in the LDB apparatus described in 2.3 for 5 min and then back in their housing room. The LDB served here as an anxiety-inducting situation, as we previously showed that this situation was required to evaluate the effects of α-CZP on the modulation of neuronal activity [16]. Ninety minutes later, the animals were culled with a lethal IP injection of sodium pentobarbital (100 mg/kg, Ceva Santé Animale, Paris, France) and were subsequently perfused as previously described [16]. The post-fixation, the cryo-protection, the freezing at −80 °C and the storage at −20 °C before sectioning of all the collected brains were carried out as described previously [16]. Coronal sections (20 µm thick) were cut using a cryostat (CM1520, Leica, Wetzlar, Germany) and stored at −20 °C until staining. Slides were rehydrated in PBS, treated with 2% BSA, and incubated with anti-c-Fos rabbit antiserum (1:5000, Ab-5, Calbiochem, Guyancourt, France) for 48 h at 4 °C. After washing with PBS, the slides were incubated with a secondary goat anti-rabbit Alexa-Fluor 488 (1:200, Molecular Probes, Illkirch, France) for 2 h at room temperature and eventually mounted using a medium containing DAPI (Vector Laboratories, Burlingame, CA, USA). The slides were digitised using either a Lamina (amygdala only, Cochin HistIM Facility; Perkin Elmer, Waltham, MA, USA) using a 20× objective lens and epifluorescence or an epifluorescence microscope (all other studied regions of the brain, Axio Imager.Z1, Zeiss, Ulm, Germany). The controls, either obtained by omitting the primary or the secondary antibody, were processed with each staining in order to check for non-specific staining. The same automated counting was performed with ImageJ [23] to evaluate the number of c-Fos neurons/0.04 mm^2^ [16]. Brain regions were identified using a stereotaxic atlas [24].

### 2.5. Statistical Analysis

Behavioural and immunohistochemical results were analysed using one-way analysis of variance (ANOVA) followed by Bonferroni post hoc tests for multiple comparisons analysis using R (Version 3.4.2, R Core Team, Vienna, Austria) [25]. All data are reported as mean ± SEM. Differences were considered to be significant at the *p* < 0.05 level.

## 3. Results

### 3.1. Anxiolytic-Like Activity of YLGYL in the LDB.

**Figure 1:**
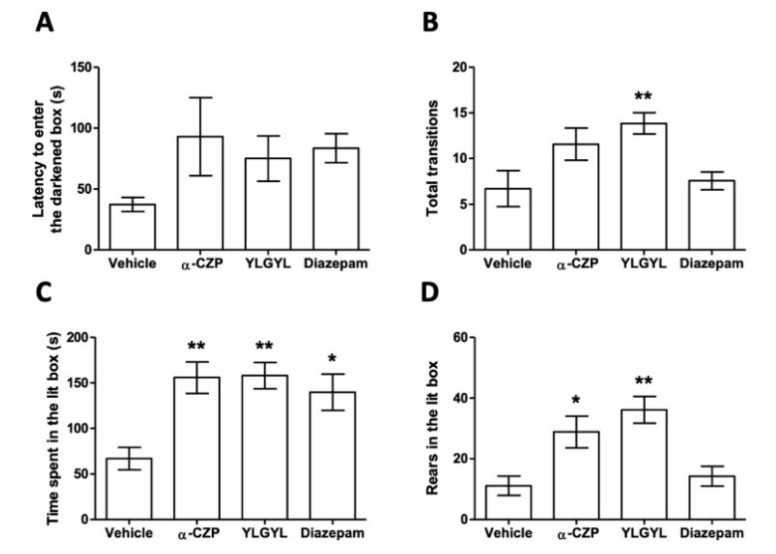
Ethological effects of α-casozepine (α-CZP), YLGYL, and diazepam on the behavioural response in the light/dark box in Swiss mice. (**A**) Latency to enter the darkened box; (**B**) Total transitions; (**C**) Time spent in the lit box; (**D**) Rears in the lit box. Mice were IP injected 30 min earlier with the vehicle, α-CZP (1 mg/kg), YLGYL (0.5 mg/kg), or diazepam (1 mg/kg). Results were analysed using a one-way analysis of variance (ANOVA) to detect the effect of the treatment on the studied behavioural scores. Subsequent comparisons were made using Bonferroni post hoc tests. Data are mean ± SEM (*n* = 7–8/group). * *p* < 0.05, ** *p* < 0.01 compared to the vehicle.

The LDB results are reported in Figure 1. An IP injection of 0.5 mg/kg of YLGYL 30 min before the test increased the number of transitions between the two compartments (F_(3,24)_ = 4.8636, *p* = 0.0177), the time spent in the lit box (F_(3,24)_ = 7.0194, *p* = 0.0034), as well as the number of rears in the lit box (F_(3,24)_ = 8.405, *p* = 0.0014), compared to a vehicle injection in the same conditions. α-CZP, injected at the same molar concentration, increased the time spent (*p* = 0.0043) and the number of rears (*p* = 0.0014) in the lit box, while diazepam only increased the time spent in the lit box (*p* = 0.0243) compared to the vehicle.

### 3.2. Modulation of Neuronal Activity induced by Intraperitoneal Injection of either YLGYL, α-CZP, or Diazepam in an Anxiety-Inducing Situation

**Figure 2:**
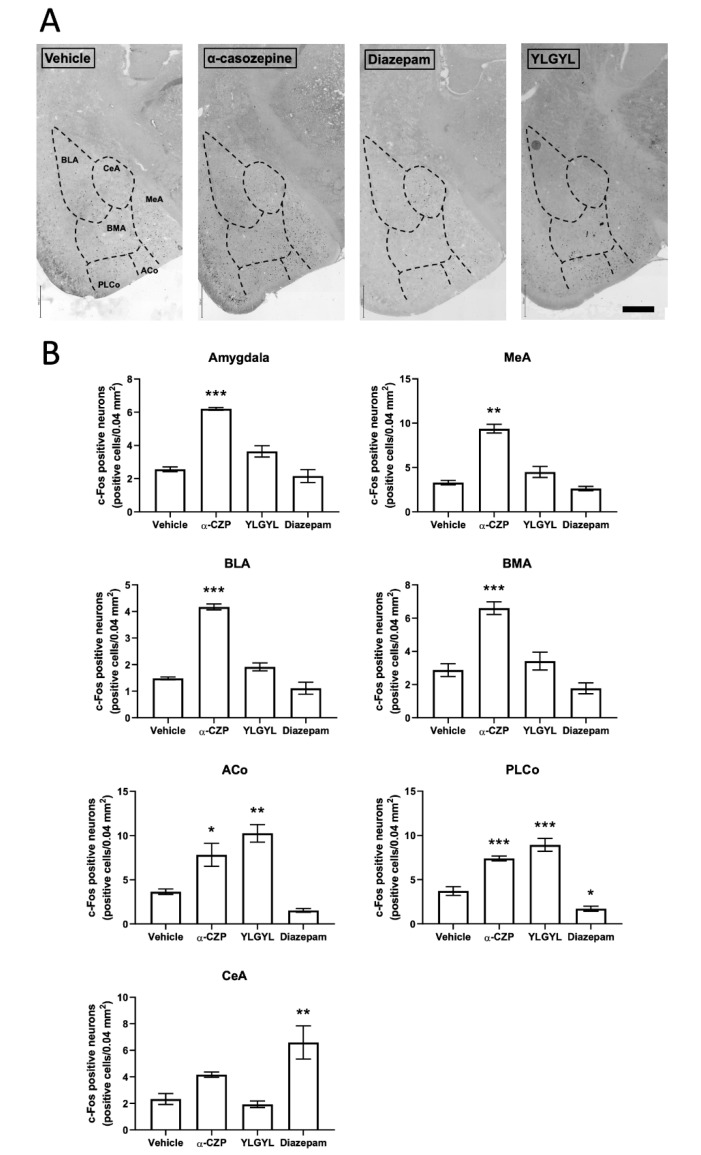
The effects of α-casozepine (α-CZP, 1 mg/kg, IP), YLGYL (0.5 mg/kg, IP), or diazepam (1 mg/kg, IP) on anxiety-induced c-Fos immunoreactivity in the amygdala. (**A**) Photomicrographs displaying the effects of the vehicle, α-CZP, YLGYL, or diazepam on anxiety-induced c-Fos immunoreactivity in the amygdala (coronal sections, bregma −1.58 mm). The green channel was extracted, and colours were inversed while the contrast was increased to 40% for better visualisation. Scale bar: 500 µm. (**B**) Corresponding densities of c-Fos immunopositive cells (displayed as number of positive cells/0.04 mm^2^). Data are mean ± SEM (*n* = 4/group). Results were analysed using a one-way analysis of variance (ANOVA) followed by a Bonferroni post hoc test to compare the effect of α-CZP, YLGYL, and diazepam to the vehicle (* *p* < 0.05, ** *p* < 0.01, *** *p* < 0.001). ACo: anterior cortical nucleus; BLA: basolateral nucleus; BMA: basomedial nucleus; CeA: central nucleus; MeA: medial nucleus; PLCo: posterolateral cortical nucleus.

An IP injection of 0.5 mg/kg of YLGYL increased c-Fos expression in the anterior and posterior cortical nuclei of the amygdala (F_(3,11)_ = 21.771, *p* = 0.0012 and F_(3,11)_ = 53.659, *p* < 0.0001, respectively, Figure 2), as well as in the raphe magnus nucleus (RMg) (F_(3,11)_ = 82.797, *p* < 0.0001, Table S1), compared to the vehicle.

An IP injection of 1 mg/kg of α-CZP increased c-Fos expression globally in the amygdala (Figure 2) (F_(3,11)_ = 51.173, *p* = 0.0002), and more specifically in the basolateral (F_(3,11)_ = 89.916, *p* < 0.0001), the basomedial (F_(3,11)_ = 28.829, *p* = 0.0013), and the medial (F_(3,11)_ = 61.17, *p* < 0.0001) nuclei, compared to the vehicle. This peptide also increased c-Fos expression in the raphe magnus nucleus compared to the vehicle (F_(3,11)_ = 82.797, *p* = 0.0006, Table S1).

**Figure 3:**
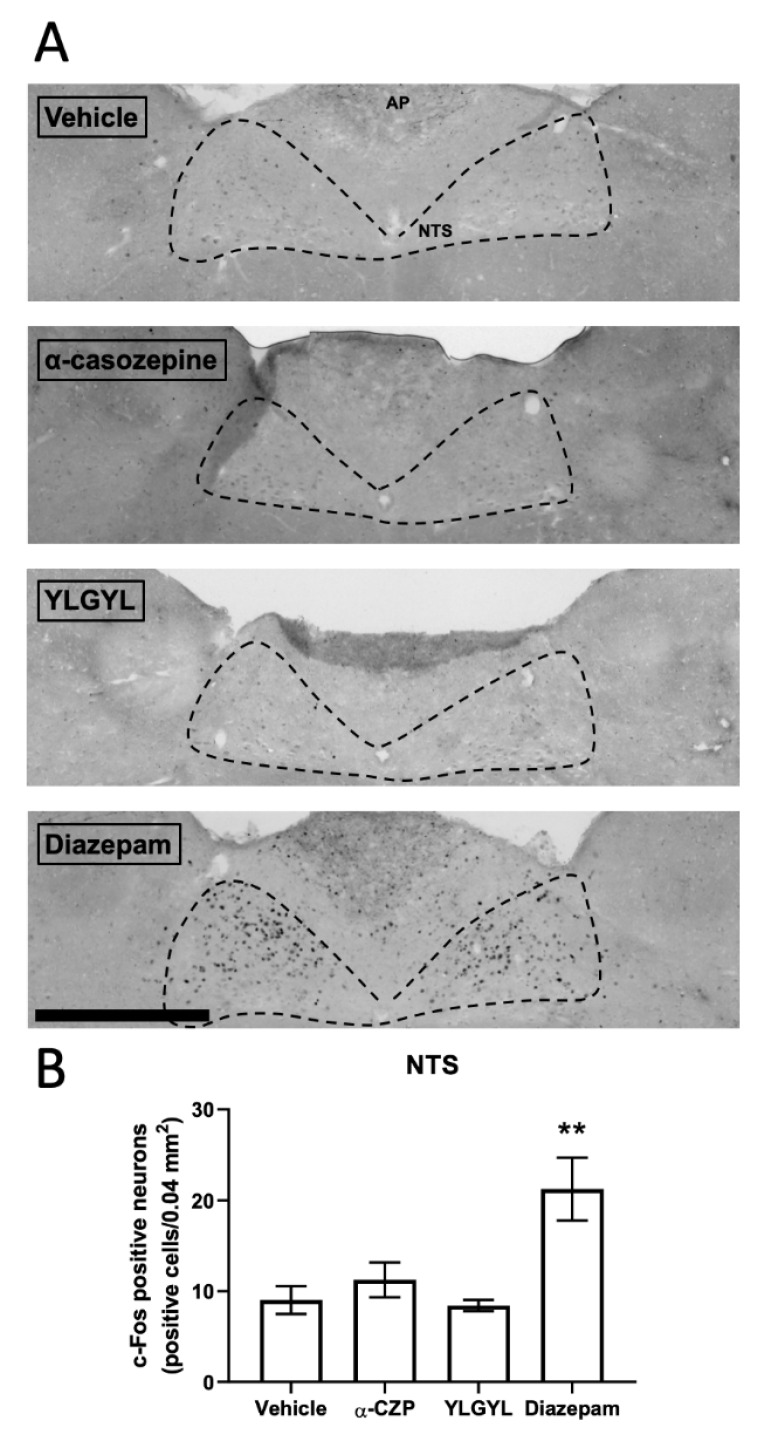
The effects of α-casozepine (α-CZP, 1 mg/kg, IP), YLGYL (0.5 mg/kg, IP), or diazepam (1 mg/kg, IP) on anxiety-induced c-Fos immunoreactivity in the NTS. (**A**) Photomicrographs displaying the effects of the vehicle, α-CZP, YLGYL, or diazepam on anxiety-induced c-Fos immunoreactivity in the NTS (coronal sections, bregma–7.56 mm). The green channel was extracted, and the colours were inversed while the contrast was increased to 40% for better visualisation. Scale bar: 500 µm. (**B**) Corresponding densities of c-Fos immunopositive cells (displayed as number of positive cells/0.04 mm^2^). Data are mean ± SEM (*n* = 4/group). Results were analysed using a one-way analysis of variance (ANOVA) followed by a Bonferroni post hoc test to compare the effect of α-CZP, YLGYL, and diazepam to the vehicle (** *p* < 0.01). AP: area postrema; NTS: nucleus of the tractus solitarius.

**Figure 4:**
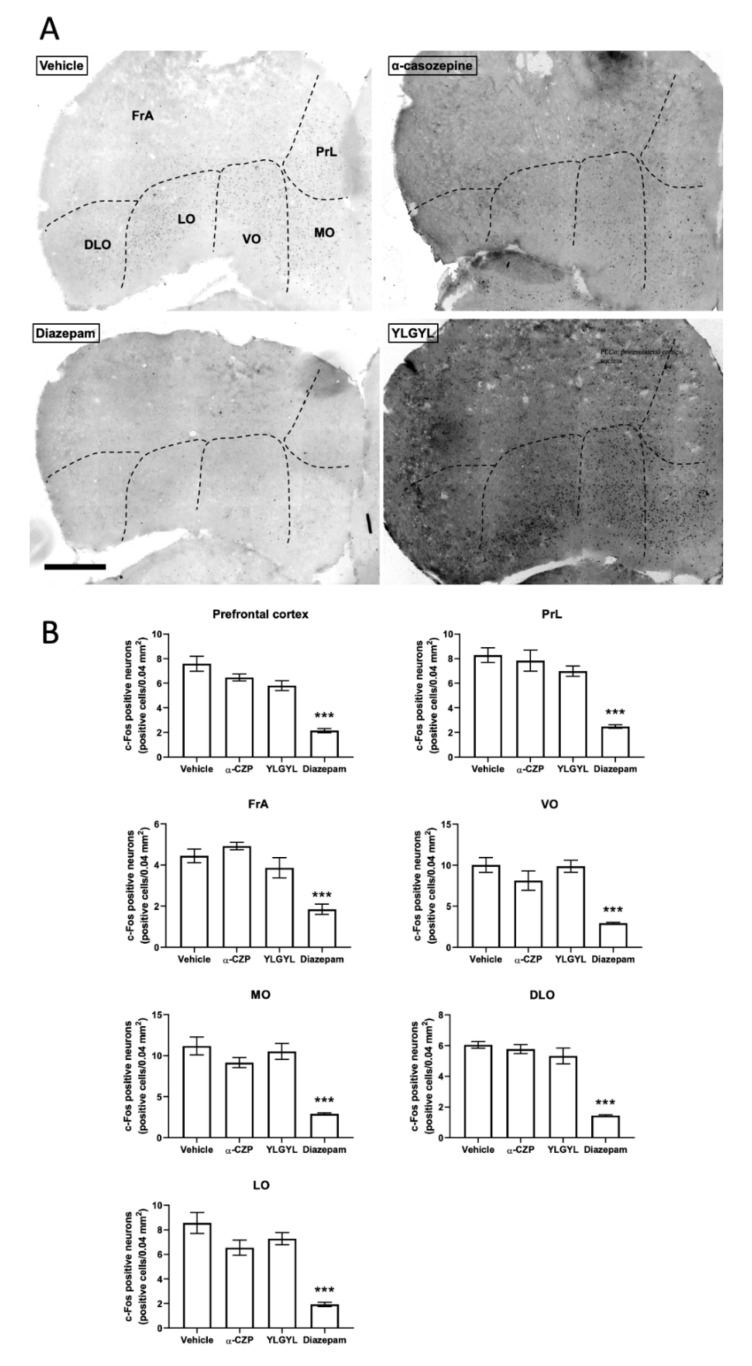
The effects of α-casozepine (α-CZP, 1 mg/kg, IP), YLGYL (0.5 mg/kg, IP), or diazepam (1 mg/kg, IP) on anxiety-induced c-Fos immunoreactivity in the prefrontal cortex. (**A**) Photomicrographs displaying the effects of the vehicle, α-CZP, YLGYL, or diazepam on anxiety-induced c-Fos immunoreactivity in the prefrontal cortex (coronal sections, bregma 2.68 mm). The green channel was extracted, and the colours were inversed while the contrast was increased to 40% for better visualisation. Scale bar: 500 µm. (**B**) Corresponding densities of c-Fos immunopositive cells (displayed as number of positive cells/0.04 mm^2^). Data are mean ± SEM (*n* = 4/group). Results were analysed using a one-way ANOVA followed by a Bonferroni post hoc test to compare the effect of α-CZP, YLGYL, and diazepam to the vehicle (*** *p* < 0.001). FrA: frontal association cortex; PrL: prelimbic cortex; DLO: dorsolateral orbital cortex; LO: lateral orbital cortex; VO: ventral orbital cortex; MO: medial orbital cortex.

Only an IP injection of 1 mg/kg of diazepam increased c-Fos expression in the nucleus tractus solitarius (NTS, Figure 3) (F_(3,11)_ = 8.5315, *p* = 0.0069) and decreased it in the prefrontal cortex (PFC, Figure 4) and periaqueductal grey (PAG, Table S1) (F_(3,11)_ = 33.262, *p* < 0.0001 and F_(3,11)_ = 6.0371, *p* = 0.0309, respectively) compared to the vehicle. This injection of diazepam also increased c-Fos expression specifically in the central nucleus of the amygdala (*p* < 0.0031).

Some differences were also observed between YLGYL and α-CZP, as YLGYL displayed a higher c-Fos expression in the raphe magnus nucleus (RMg) compared to α-CZP (*p* = 0.0001, Table S1).

## 4. Discussion

α-CZP is a peptide obtained after the tryptic hydrolysis of bovine α_s1_-casein, one of the major proteins in bovine milk. Previous studies have documented that the anxiolytic-like properties of α-CZP comparable are to those of benzodiazepines in rodents [1,15,16]. Recently, it has been shown to modulate neuronal activity in mice after intraperitoneal injection [16]. The tryptic hydrolysate containing α-CZP shows anxiolytic activity in several species, including humans, after oral administration. α-CZP does not need any modification by the proteolytic enzymes of the digestive tract to display its activity, as it is active after IP injection. Nevertheless, it cannot be excluded that after its ingestion, followed by the digestion process, other fragments may carry the anxiolytic activity, and take part in the overall anxiolytic action and allow the crossing of physiological barriers. It is interesting to wonder about the peptides that can be formed after the direct ingestion of α_s1_-casein. α-CZP was found in the stomachs of mini-pigs 4 min after the ingestion of raw milk [26]. Peptides from the 90–100 region of α_s1_-casein have also been identified in the proximal and distal jejunum of mini-pigs after skimmed milk powder ingestion [27]. In humans, the peptides YLGYLEQ and YLGYEQLL have been detected in jejunal effluents after casein consumption [28]. The peptide YLGYLEQ demonstrates anxiolytic activity in rats [14]. No peptide from region 91–100 region of α_s1_-casein was identified by Sanchón and colleagues, but the hydrolysis of peptide bonds 89–90 and 100–101 are confirmed, since peptides 85–89 and 101–112 were identified [29]. However, differences in peptide genesis could exist depending on the matrix of administration [26] and interindividual variability, especially concerning pepsin and the trypsin/chymotrypsin ratio [30]. The proteolysis of α-CZP has only been studied in vitro. After an in vitro hydrolysis of α-CZP by either Corolase PP^®^ or by pepsine followed by Corolase PP^®^, the peptides YLGYLEQ and YLGLY are the main fragments in the hydrolysis medium with a proportion of the pentapeptide higher than 20% [14]. The peptide YLGYL is found after hydrolysis by pepsin in vitro, followed by trypsin or elastase of the synthetic fragment 86–100 of bovine α_s1_-casein [15], and this peptide is also found after 30 min in the gastric phase of a dynamic in vitro digestion model of milk proteins [27]. We chose to check whether or not this peptide retained the anxiolytic activity of α-CZP, and if it was the case, to compare the activity levels and modulations of neuronal activity of both peptides. Unlike peptides 91–96, 91–97, 91–98 or 91–99, YLGYL displays a less acidic pHi, close to that of α-CZP. In addition, it possesses the two tyrosine residues which could mimic the structure of nitrazepam, a benzodiazepine [31], unlike the synthetic shorter peptides YL and YLG, of which anxiolytic activity is also shown in mice [15,32]. These two peptides, with sequences present in the α-CZP sequence, also have anxiolytic properties, but in contrast to α-CZP [1], their activity does not involve the benzodiazepine site of the GABA_A_ receptor [15,32,33]. The anxiolytic-like properties of both YL and YLG were blocked by antagonists for serotoninergic 5-HT_1A_, dopamine D_1_, and GABA_A_ receptors without a direct agonist action on these receptors [15,32]. Comparing whether the anxiolytic activity of YLGYL, that contains both the YL and YLG sequences, is close or not to that of α-CZP, could allow progress in the knowledge of the peptides from which they are derived.

The present results showed, for the first time, that the shorter N-terminal peptide, YLGYL, derived from α-CZP, also displayed the same anxiolytic-like activity. Indeed, an IP injection of 0.5 mg/kg (corresponding to 0.8 μmol/kg) YLGYL in male Swiss mice induced an anxiolytic-like activity in an LDB paradigm. The reduction in the time spent in the dark area, which is an indicator of the anxiolytic-like activity of a molecule [34], is similar to both α-CZP injected at the same molar concentration, and diazepam. This agrees with the proposed role of the two tyrosyl residues of the N-terminal sequence of α-CZP in the active structure of the peptides [14,31]. Although longer, α-CZP can exert its anxiolytic-like properties because the C-terminal part of this peptide may allow the maintenance of a suitable active conformation of the pattern, implying the tyrosyl residues despite the constraints caused by the addition of residues. The decapeptide α-CZP was screened by its affinity on the benzodiazepine site of GABA_A_ receptors [1]. This action on GABA_A_ receptors by the active peptide of α_s1_-casein tryptic hydrolysate is supported by the fact that this hydrolysate displays a direct modulation on these receptors [10], and that it affects protein levels of GABA_A_ receptor subtypes in hypothalamic neurons [35]. The loss of the YXXY pattern in a peptide could then mean a potential loss of the benzodiazepine site affinity, as the distance between the centres of the two tyrosine aromatic rings has been shown to be similar to that between the centres of the aromatic rings in nitrazepam [31]. Moreover, the loss of this pattern could also mean a potential loss of affinity for opioid receptors, as the ArXXAr (where Ar is an aromatic residue) pattern seems to be crucial for the affinity of opiate peptides [36]. As such, the anxiolytic-like activity of the shorter dipeptide YL was also shown not to be reliant on opioid receptors [32]. As YLGYL shows some similarities with RYLGYL, α_s1_-casein-(f90–95), which has some affinity for opioid receptors [37], an involvement of the opioid system in the mode of action of YLGYL could have been hypothesised. However, Loukas et al. concluded that YLGYL is relatively inactive to the tested opioid receptors, especially compared to RYLGYLE, α_s1_-casein-(f90–96) [37]. In summary, it could then be hypothesised that all the peptides derived from α-CZP, although all being anxiolytic, may not mediate their action by similar ways in terms of the target receptors or neurotransmitters involved and may not have the same bioavailability.

A single IP injection of 1 mg/kg of α-CZP induced changes in c-Fos expression in different brain regions after a challenge by an anxiety-inducing situation (i.e., the LDB). Anxious or stressful situations previously appeared to be mandatory for highlighting the anxiolytic-like activity of the tryptic hydrolysate of bovine α_s1_-casein [7,9,13] and of α-CZP [16]. In this anxiety-inducing situation, after a single IP injection of 1 mg/kg of α-CZP, c-Fos expression decreased in the hippocampal formation, accumbens nucleus, dorsomedial, paraventricular and ventromedial nuclei of the hypothalamus, and bed nucleus of the stria terminalis (BNST), and increased in the amygdala [16]. The present study confirmed the absence of effect in the prefrontal cortex and the increase in c-Fos expression in the amygdala (more specifically in the basolateral, basomedial, medial, and posterolateral cortical nuclei, and at a lower level in the anterior cortical nucleus) after the injection of α-CZP. Benoit et al. also showed, and it was reinforced in this study, that an IP injection of diazepam had no effect on global c-Fos expression in the amygdala, while it decreased this expression in the prefrontal cortex [16]. The new results obtained with this work in the NTS, PAG, and RMg also pointed out some differences between α-CZP, that increased c-Fos expression in the RMg, and diazepam, that increased c-Fos expression in the NTS and decreased it in the PAG. These three regions located in the brainstem are also linked to anxiety. The NTS serves as the relay of vagal afferences to the brain, and diazepam-sensitive GABA_A_ receptors have been characterised in this region [38,39]. The PAG is the structure associated with freezing and escape behaviours [40]. The RMg belongs to the raphe nuclei system which innervates the forebrain with serotoninergic projections [41]. These differences are in line with a different mode of action between α-CZP and diazepam, which may explain the absence of side effects of the tryptic hydrolysate containing α-CZP [2,10].

The IP injection of 0.5 mg/kg YLGYL also impacted c-Fos expression in some of the same regions as α-CZP, with an increase in c-Fos expression in the anterior cortical and posterolateral cortical nuclei of the amygdala and in the RMg. It appeared that YLGYL affects a subset of the cortical nuclei and not the basolateral, basomedial, and medial nuclei of amygdala, which contrariwise responded to the administration of α-CZP. Diazepam did not modify global c-Fos expression in the amygdala, but it nevertheless increased this expression in the central nucleus. This observation was consistent with previous works, since diazepam intraperitoneally injected at 1 mg/kg increases c-Fos expression in the amydgala only in the lateral division of the central nucleus [42]. Chlordiazepoxide, another benzodiazepine, also increases c-Fos expression in the central nucleus of the amydgala [43]. Linking the effects of an anxiolytic molecule from its natural source to the expression of c-Fos in the amygdala may be complex. Indeed, vetiver essential oil increases c-Fos expression in the lateral division of the central nucleus but not in the other parts of amygdala [42]. An increase in c-Fos expression in the central nucleus of the amygdala is also highlighted with lavender oil [43]. The repeated administration of yokukansan followed by an acute restraint stress decreases the stress-induced increase in c-Fos expression in basolateral and medial amygdaloid nuclei, but not in the central amygdaloid nucleus [44]. α-CZP increased c-Fos expression in all the studied parts of amygdala but the central nucleus, and YLGYL only in the anterior cortical and posterolateral cortical nuclei. Within the amygdala, balanced opposite pathways regulate anxiety, as subpopulations or projections of neurons belonging to the basolateral amygdala can act in opposition in their connections to the central amygdala. In addition, the involvement of the amygdala in anxiety cannot exclude other parallel circuits [45]. The increase in c-Fos expression in the RMg was significantly higher with YLGYL than with α-CZP. These differences between α-CZP and YLGYL may be insignificant, but they may also be interpreted as differences in the ways in which α-CZP and YLGYL may modulate anxiety, since the shorter peptides, YL and YLG, seem to act differently. Although they derive from the same sequence, all of these peptides act in a more or less different way but participate in a global anxiolytic action.

## 5. Conclusions

The goal of this study was to evaluate the anxiolytic-like properties of YLGYL, a peptide fragment released during the in vitro digestion of α-CZP, a food-derived bioactive peptide displaying anxiolytic-like properties, and that could be found after in vivo hydrolysis of α_s1_-casein. The effects of both of these peptides on the modulation of neuronal activation in brain regions involved in anxiety regulation was also evaluated. We demonstrated that a single IP injection of 0.5 mg/kg YLGYL also exhibits anxiolytic-like properties in Swiss mice using the LDB paradigm at a molar concentration similar to that of α-CZP. The effect of α-CZP on neuronal activity in the prefrontal cortex and amygdala while animals were put in an anxiety-inducing situation, was confirmed in this study, while some new differences in the nucleus of the tractus solitarius, periaqueductal grey, and raphe magnus nucleus were spotted between the peptide and a reference benzodiazepine, diazepam. The administration of YLGYL had also quite similar effects as α-CZP on neuronal activity, compared to the vehicle and diazepam in the same situation. Some differences in the amygdala and raphe nuclei were still pointed out between the two peptides and further pharmacological studies would help understand the mode of action of all these food-derived anxiolytic-like peptides.

## Supplementary Materials

The following are available online at https://www.mdpi.com/2072-6643/12/5/1497/s1, Table S1: Effects of an i.p. injection of α-casozepine (α-CZP), YLGYL, and diazepam on the anxiety-induced c-Fos immunoreactivity (positive cells/0.04 mm^2^) in different areas of mice brains (*n* = 4/group).
