# Supplementary material for: Anxiolytic Activity and Brain Modulation Pattern of the α-Casozepine-Derived Pentapeptide YLGYL in Mice
Source: Nutrients. 2020 May 21;12(5):1497. doi: 10.3390/nu12051497 (PMC7285003; doi:10.3390/nu12051497)
Supplement: Supplementary file 1 [file nutrients-12-01497-s001.pdf]

Table S1. Effects of an i.p. injection of α-casozepine (α-CZP), YLGYL, and diazepam (on the anxiety-induced c-Fos immunoreactivity (positive cells/0.04 mm^2) in different areas of mice brains (n = 4/group). Mice were injected with either vehicle, α-CZP (1 mg/kg), YLGYL (0.5 mg/kg), or diazepam (1 mg/kg) 90 mins before culling. Data are mean ± SEM. Results were analysed using a one-way ANOVA to detect the effects of treatment (*p < 0.05, **p < 0.01, ***p < 0.001) and a Bonferroni post-hoc test was performed to compare the effect of α-CZP, YLGYL and diazepam to the vehicle (†p < 0.05, ††p < 0.01, †††p < 0.001).

	**Vehicle**	**α-CZP**	**YLGYL**	Diazepam	ANOVA	
					$F_{(3,11)}$	(p-value)
**Prefrontal cortices**						
Global	7.59 ± 0.61	6.47 ± 0.27	5.81 ± 0.41	2.15 ± 0.16 †††	33.262	8.21E-06 ***
Frontal association cortex	4.45 ± 0.33	4.92 ± 0.18	3.86 ± 0.49	1.85 ± 0.24 †††	14.661	3.69E-04 ***
Prelimbic cortex	8.28 ± 0.60	7.84 ± 0.85	6.98 ± 0.42	2.49 ± 0.14 †††	27.389	2.11E-05 ***
Medial orbital cortex	11.18 ± 1.09	9.15 ± 0.60	10.52 ± 0.96	2.91 ± 0.13 †††	22.654	5.19E-05 ***
Ventral orbital cortex	10.03 ± 0.90	8.12 ± 1.19	9.87 ± 0.74	2.96 ± 0.09 †††	19.242	0.00011 ***
Lateral orbital cortex	8.56 ± 0.85	6.54 ± 0.62	7.28 ± 0.50	1.83 ± 0.17 †††	25.734	2.84E-05 ***
Dorsolateral orbital cortex	6.05 ± 0.23	5.77 ± 0.29	5.32 ± 0.52	1.45 ± 0.05 †††	45.99	1.63E-06 ***
**Amygdala**						
Global	2.56 ± 0.14	6.21 ± 0.08 †††	3.64 ± 0.34	2.16 ± 0.39	51.173	9.47E-07 ***
Anterior cortical nucleus	3.65 ± 0.31	7.82 ± 1.30 †	10.25 ± 1.00 ††	1.54 ± 0.21	21.771	6.25E-05 ***
Posterolateral cortical nucleus	3.71 ± 0.49	7.39 ± 0.29 †††	8.93 ± 0.73 †††	1.72 ± 0.29 †	53.659	7.43E-07 ***
Basolateral nucleus	1.49 ± 0.05	4.17 ± 0.11 †††	1.91 ± 0.15	1.11 ± 0.23	89.916	5.07E-08 ***
Basomedial nucleus	2.88 ± 0.39	6.60 ± 0.38 †††	3.41 ± 0.54	1.78 ± 0.33	28.829	1.65E-05 ***
Central nucleus	2.33 ± 0.41	4.17 ± 0.20	1.93 ± 0.25	6.59 ± 1.25 ††	11.663	0.00133 **
Medial nucleus	3.29 ± 0.26	9.38 ± 0.49 ††	4.48 ± 0.62	2.63 ± 0.24	61.17	3.79E-07 ***
**Nucleus of the Tractus Solitarius**	9.03 ± 1.54	11.25 ± 1.90	8.42 0.64	21.24 ± 3.45 ††	8.5315	0.00328 **
**Periaqueductal Grey**	6.68 ± 0.65	6.01 ± 0.96	7.03 ± 0.86	3.13 ± 0.53 †	6.0371	0.01101 *
**Raphe magnus nucleus**	1.61 ± 0.17	3.18 ± 0.05 †††	5.35 ± 0.43 †††	1.20 ± 0.08	82.797	7.83E-08 ***
